# EuCARE-POSTCOVID Study: a multicentre cohort study on long-term post-COVID-19 manifestations

**DOI:** 10.1186/s12879-023-08595-0

**Published:** 2023-10-13

**Authors:** Benedetta Varisco, Francesca Bai, Sara De Benedittis, Alessandro Tavelli, Alessandro Cozzi-Lepri, Matteo Sala, Federica Gaia Miraglia, Maria Mercedes Santoro, Francesca Ceccherini-Silberstein, Yishai Shimoni, Sivan Ravid, Tal Kozlovski, Florian König, Nico Pfeifer, Elham Shamsara, Milosz Parczewski, Antonella d’Arminio Monforte, Francesca Incardona, Chiara Mommo, Giulia Marchetti

**Affiliations:** 1 Department of Health Sciences, Clinic of Infectious Diseases, University of Milan, ASST Santi Paolo E Carlo, Via A Di Rudinì 8, 20142 Milan, Italy; 2 ICONA Foundation, Milan, Italy; 3 Centre for Clinical Research, Epidemiology, Modelling and Evaluation (CREME), Institute for Global Health, UCL, London, UK; 4 Clinical Trials and Grant Office, ASST Santi Paolo E Carlo, Milan, Italy; 5 Department of Experimental Medicine, University of Rome Tor Vergata, Rome, Lazio Italy; 6 Healthcare Informatics, IBM Research-Haifa, Mount Carmel Haifa, Israel; 7 Institute for Bioinformatics and Medical Informatics, University of Tübingen, Tübingen, Germany; 8 Department of Infectious Tropical Diseases and Immune Deficiency, Pomeranian Medical University in Szczecin, Szczecin, Poland; 9 IPRO-InformaPRO S.R.L, Rome, Italy; 10 EuResist Network, Rome, Italy

**Keywords:** Post-COVID-19 condition, Post Acute Sequelae of SARS CoV-2 infection, Long COVID, Residual organ damage in COVID-19, Follow-up after COVID-19, Persistence of COVID-19 symptoms, Gender association in post-COVID-19, Variant association in post-COVID-19

## Abstract

**Background:**

Post-COVID-19 condition refers to persistent or new onset symptoms occurring three months after acute COVID-19, which are unrelated to alternative diagnoses. Symptoms include fatigue, breathlessness, palpitations, pain, concentration difficulties ("brain fog"), sleep disorders, and anxiety/depression. The prevalence of post-COVID-19 condition ranges widely across studies, affecting 10–20% of patients and reaching 50–60% in certain cohorts, while the associated risk factors remain poorly understood.

**Methods:**

This multicentre cohort study, both retrospective and prospective, aims to assess the incidence and risk factors of post-COVID-19 condition in a cohort of recovered patients. Secondary objectives include evaluating the association between circulating SARS-CoV-2 variants and the risk of post-COVID-19 condition, as well as assessing long-term residual organ damage (lung, heart, central nervous system, peripheral nervous system) in relation to patient characteristics and virology (variant and viral load during the acute phase). Participants will include hospitalised and outpatient COVID-19 patients diagnosed between 01/03/2020 and 01/02/2025 from 8 participating centres. A control group will consist of hospitalised patients with respiratory infections other than COVID-19 during the same period.

Patients will be followed up at the post-COVID-19 clinic of each centre at 2–3, 6–9, and 12–15 months after clinical recovery. Routine blood exams will be conducted, and patients will complete questionnaires to assess persisting symptoms, fatigue, dyspnoea, quality of life, disability, anxiety and depression, and post-traumatic stress disorders.

**Discussion:**

This study aims to understand post-COVID-19 syndrome's incidence and predictors by comparing pandemic waves, utilising retrospective and prospective data. Gender association, especially the potential higher prevalence in females, will be investigated. Symptom tracking via questionnaires and scales will monitor duration and evolution. Questionnaires will also collect data on vaccination, reinfections, and new health issues. Biological samples will enable future studies on post-COVID-19 sequelae mechanisms, including inflammation, immune dysregulation, and viral reservoirs.

**Trial registration:**

This study has been registered with ClinicalTrials.gov under the identifier NCT05531773.

**Supplementary Information:**

The online version contains supplementary material available at 10.1186/s12879-023-08595-0.

## Introduction

The coronavirus disease 2019 (COVID-19) pandemic has caused widespread morbidity and mortality worldwide, with over 5 million deaths attributed to the acute syndrome caused by the severe acute respiratory syndrome coronavirus 2 (SARS-CoV-2) virus [1].

However, the long-term consequences of COVID-19, also known as post-acute sequelae of COVID-19 (PASC) or long-COVID, are also concerning [2–4]. These long-lasting symptoms may include fatigue, muscle weakness, shortness of breath, cough, joint pain, chest pain, anosmia or dysgeusia, cognitive dysfunctions, sleep disorders, anxiety and depression. These symptoms may be new, following initial recovery from the acute COVID-19 illness, or may persist from the beginning of the disease. In October 2021, the World Health Organization (WHO) identified the “post COVID-19 condition” as the presence of symptoms usually three months after the onset of COVID-19 that last for at least two months and cannot be explained by a different diagnosis. Patients may also be diagnosed with residual organ damage after acute COVID-19, particularly in the lungs, heart, central nervous system, and peripheral nervous system.

Preliminary data have shown a high prevalence of post-COVID-19 condition, although there is considerable variation among different studies. This variation can be attributed to the utilisation of different definitions for post-COVID-19 condition, inclusion of diverse populations, and the use of varying or non-standardised methods to assess the persistence of symptoms.

A recent systematic review of 20 observational studies, comprising 5,440 enrolled patients, reported a prevalence of post-COVID-19 condition between 4.7% to 80% [5]. Several studies have demonstrated high rates of ongoing symptoms after hospital discharge for acute COVID-19 illness (87% after two months and 70% after six months) and in outpatient COVID-19 subjects (32% at 30–45 days from the acute episode) [6–8]. A systematic review of 57 studies, comprising more than 250,000 patients recovered from COVID-19, showed that 54% of patients reported at least one post-acute sequelae at one month (short-term), 55% at 2–5 months (intermediate term), and 54% at 6 or more months (long-term) [9].

As previously mentioned, identifying risk factors for this long-term condition is a challenging task due to the abundance of studies conducted in various settings and populations. There is conflicting evidence regarding the possible association between disease severity during the acute phase and the risk of developing post-COVID-19 condition [10–12]. Similarly, there is no consensus on the possible role of several other factors, such as acute symptoms, female gender [13], older age, smoking, body mass index, and pre-existing comorbidities, on development of SARS-CoV-2 sequelae.

The possible predictors of post-COVID-19 condition include the severity of disease, the presence of hyperinflammation or autoimmune responses, and persistent viremia during the acute phase.

Additionally, viral variants may differently affect post-COVID-19 condition, with the effect of new variants on transmission rate, immune evasion, and incidence of post-acute sequelae still to be understood [14].

Several indirect effects of acute COVID-19 could also affect post-COVID-19 condition, such as a long time of hospitalisation and home isolation, economic damage and loss of job.

## Methods and analysis

### The EuCare Project

The European cohorts of patients and schools to advance response to epidemics (EuCARE) project is a collaborative effort between several cohorts of patients and schools across various geographic regions, including European and extra-European countries (www.eucareresearch.eu). The project aims to provide an advanced response to COVID-19 epidemics by consolidating or expanding interactions among different clinical and research centres.

The project brings together a comprehensive, multidisciplinary team of clinicians, virologists, epidemiologists, statisticians, and experts in machine-learning. The primary aim of the study is to examine various facets of the COVID-19 pandemic. These include studying natural and vaccine-induced immunity to different viral variants among healthcare workers, tracking the clinical progress and long-term outcomes of COVID-19 patients admitted to hospitals, and identifying effective strategies to assist schools during similar COVID-19 outbreaks [15]. One of the key areas of investigation will be the post COVID-19 condition.

As part of the EuCARE project, Work Package 3 (WP3) has been designed to investigate the impact of COVID-19 on both hospitalised and outpatient populations. WP3 includes two distinct cohorts: the EuCARE-HOSPITALISED Study, which is currently collecting data from COVID-19 patients during the acute phase of hospitalisation, and the EuCARE POSTCOVID Study, whose aim is to gather data on post-COVID-19 conditions from both hospitalised and outpatient COVID-19 patients. The focus of this paper is the EuCARE-POSTCOVID Study, and we present its protocol here.

### Study hypotheses

The main EuCARE-POSTCOVID Study hypothesis is that a significant proportion of patients who recover from acute COVID-19 will experience long-term sequelae characterised by the presence of physical and/or psychological symptoms persisting for at least three months after the acute illness. Initial evidence suggests that patients experiencing persistent symptoms at 4–8 weeks after acute disease were more likely to be elderly, female, and hospitalised during the acute phase [16], in line with this EuCARE-POSTCOVID Study which also hypothesises that older age, female gender, higher severity of disease, and patients' comorbidities could be risk factors for the development of post COVID-19 condition.

Finally, the study hypothesis is that the Omicron variant of SARS-CoV-2 could be associated with lower incidence of post-COVID-19 condition, possibly as a reflection of lower inflammation and disease severity during the acute phase of disease. Moreover, the type and breakthrough infectivity rates of SARS-CoV-2 variants among fully vaccinated individuals may modify the incidence and manifestation of post-COVID-19 condition [9, 17]. Thus, the evaluation of circulating SARS-CoV-2 variants is crucial in predicting their potential association with post-COVID-19 condition.

### Study objectives

The primary objective of the study is to assess the incidence and risk factors of post-COVID-19 condition in a cohort of recovered COVID-19 patients.

The secondary objectives of the study are as follows:

- To evaluate the association between circulating SARS-CoV-2 variants and the risk of post-COVID-19 condition.
- To identify different clinical phenotypes of post-COVID-19 condition.
- To evaluate the long-term residual organ impairment, specifically of the lung, heart, central nervous system, and peripheral nervous system, in relation to patient demographic and clinical characteristics such as age, gender, comorbidities, severity of disease, and virology including viral variant, specific mutations, and viral load in the acute phase.
- To compare the incidence of post COVID-19 condition between males and females.

## Expected outcomes

The expected outcomes of this study include:

- Determination of the overall incidence of post-COVID-19 condition in the study cohort.
- Identification of patients who continue to experience symptoms beyond 2–3 months from the acute COVID-19 disease.
- Identification of cluster of symptoms that identify different clinical phenotypes of post-COVID-19 condition.
- Identification of predictors of post-COVID-19 condition, such as demographic, clinical, and virological factors.
- Evaluation of long-term organ damage associated with post COVID-19 condition, specifically in the lungs, heart, central nervous system, and peripheral nervous system.
- Characterisation of the virological and immunological phenotype of post-COVID-19 condition, which can provide insight into the mechanisms underlying this condition.

### Study design

This is an observational retrospective and prospective, multicentre, cohort study. The study will last for 3 years, from February 2022 to February 2025.

### Study population

The study will enrol adult patients (> 18 years old) diagnosed with acute COVID-19 disease between 01/03/2020 and 01/02/2025 at the participating centres. The COVID-19 patients will be divided into two groups: Group 1 (hospitalised) and Group 2 (outpatients).

Inclusion criteria for the study require a confirmed diagnosis of SARS-CoV-2 infection based on a positive SARS-CoV-2 RNA test on nasopharyngeal swab or other respiratory samples. Mild COVID-19 disease is defined as cases without hospital admission, while moderate/severe disease includes cases requiring hospitalisation for COVID-19 or hospitalisation for other medical issues with a positive SARS-CoV-2 RNA sample. Patients must provide informed consent for the study to be eligible for inclusion.

Exclusion criteria include death during hospitalisation and patient refusal to participate.

**Fig. 1:**
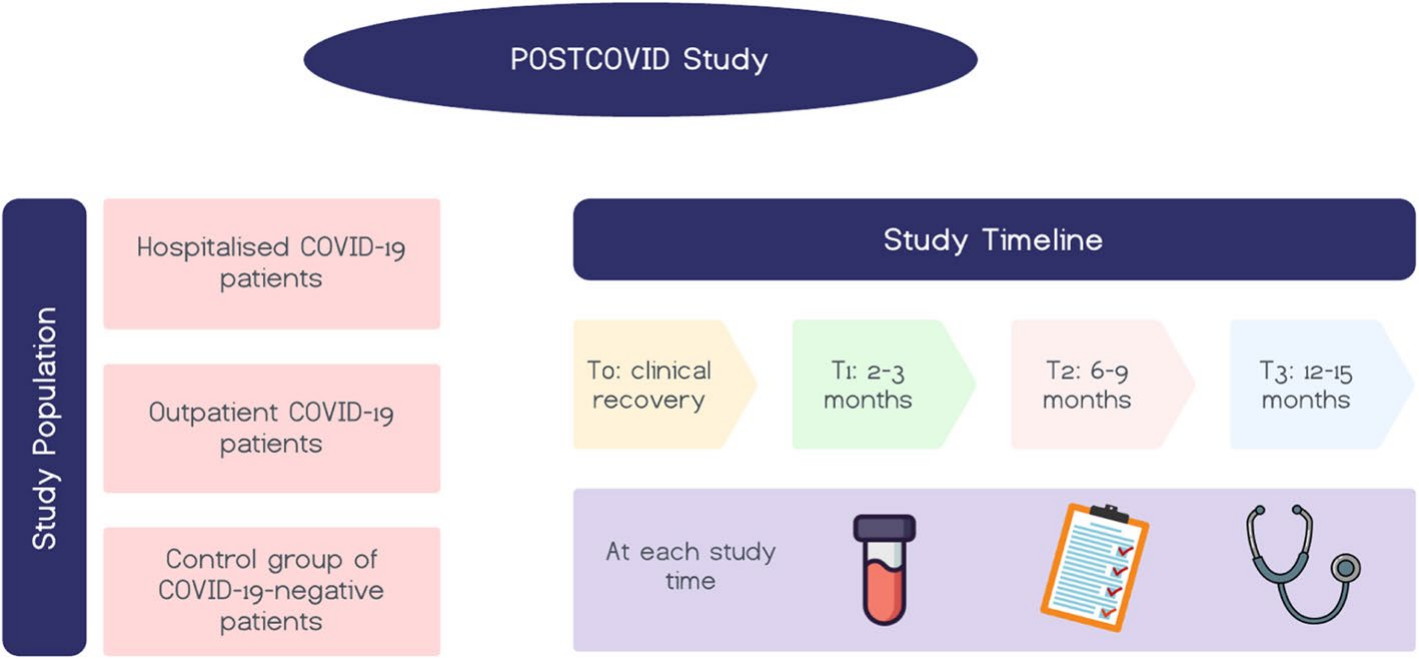
Depicting the study population and timeline

The study will also enrol a control group of hospitalised patients for respiratory infections other than COVID-19 disease during the same study period, with enrolment occurring at hospital discharge and follow-up appointments scheduled at the post COVID clinic with the same timing as COVID-19 patients. These patients will be enrolled if they are adults hospitalised for respiratory infections requiring any type of oxygen therapy or Intensive Care admission (Fig. 1).

### Study centres

The coordinator centre is the Clinic of Infectious Diseases, San Paolo Hospital, ASST Santi Paolo and Carlo, Department of Health Sciences, University of Milan, Italy. Other participating centres are Vilnius University Hospital, Santaros Klinikos, Vilnius, Lithuania; University Hospital Heinrich Heine of Dusseldorf, Dusseldorf, Germany; Policlinico Tor Vergata, Università degli Studi di Roma Tor Vergata, Rome, Italy; Regional Hospital Dr. Juan Graham Casasús, Villahermosa, Tabasco, Mexico; Centro Hospitalar de Lisboa Ocidental, Lisbon, Portugal; Pomeranian Medical University, Szczecin, Poland and Federal University of Minas Gerais, Minas Gerais, Brazil.

### Patient assessment and follow-up protocol

The patient assessment and follow-up protocol for acute COVID-19 disease involves follow-up visits at 2–3, 6–9, and 12–15 months after clinical recovery. At each visit, routine blood exams and medical visits will be conducted, along with several assessments via questionnaires, including the short version of the post COVID-19 WHO Case Report Form (WHO CRF) for collection of symptoms and newly emerged complaints after COVID-19, the Fatigue Numerical Rating Scale, the Medical Research Council (MRC) dyspnoea scale, the 5-level EQ-5D test (EQ5D-5L), the 12-item WHO Disability Assessment Schedule (WHODAS), the Montreal Cognitive Assessment (MOCA), the Hospital Anxiety and Depression Scale (HADS-A/D), and a screening tool named PCL-5 for Post-Traumatic Stress Disorder (PTSD). If patients are unable to attend the post-COVID-19 clinic, the questionnaires will be able to be completed through a phone interview. All questionnaires will be standardized and available in multiple languages (Fig. 1).

Further instrumental diagnostic evaluations such as diffusing capacity of lung carbon monoxide (DLCO), spirometry, lung computed tomography (CT) scans, pneumological evaluation, echocardiography, cardiological evaluation, neurological evaluation, rehabilitation medicine specialist examination, and psychological evaluation will be conducted as necessary.

Pulmonary symptoms are common after the acute phase, thus patients undergo the MRC dyspnoea scale and oxygen saturation measurement at each post-COVID visit. Further evaluations such as chest CT scans, diffusing capacity of lung carbon monoxide (DLCO), spirometry, and a complete pneumological evaluation will be performed if oxygen saturation is < 95% or MRC dyspnoea scale grade is 2–5.

Patients who recovered from acute COVID-19 disease could present a higher risk of cardiac diseases, including heart failure, myocardial infarction, and arrhythmia in the mid- and long-term [18]. During the scheduled post-COVID visits, patients undergo a complete medical examination, including heart rate and blood pressure measurement, and fill in the MRC dyspnoea scale. Further evaluations such as an electrocardiogram, a cardiologic visit, and an echocardiography will be performed as necessary.

Neurocognitive and neuropsychiatric sequelae are important to investigate for their great impact on patients’ quality of life [6, 19, 20]. Neurocognitive and neuropsychological performance will be assessed by questionnaires, including the previously mentioned HADS-A/D, PCL-5, and MOCA, which will be performed at each scheduled visit. Patients will be referred to a psychologist or a neurologist as necessary (Table 1).

**Table 1:** Displaying the specific assessments to be conducted at each study time point

Study Flowchart				
**Procedures**	**At acute phase**	**At T1 (2–3 months)**	**At T2 (6–9 months)**	**At T3 (12–15 months)**
Nasopharyngeal swab	X			
Demographic/clinical data collection	X			
Routine blood tests		X	X	X
Blood/serum samples collection		X	X	X
Medical visit, oxygen saturation (SO2) measurement		X	X	X
Symptoms assessment (WHO CRF)		X	X	X
Fatigue Numerical Rating and Severity Scale		X	X	X
Modified MRC Dyspnoea Scale		X	X	X
Hospital re-admission, relapse or new comorbidity		X	X	X
COVID-19 vaccination status		X	X	X
QoL (EQ5D-5L)		X	X	X
Montreal Cognitive Assessment (MOCA)		X	X	X
Psychological questionnaires (HADS-A/D, PCL-5)		X	X	X
WHODAS questionnaire		X	X	X
If abnormal SO2, DLCO		X		X
If abnormal SO2, spirometry		X		X
If abnormal SO2 and/or clinically indicated, pneumology visit		X	X	X
Electrocardiogram (ECG)		X		
If cardiac symptoms, echocardiogram		X		X
If cardiac symptom and/or clinically indicated, cardiology visit		X	X	X
If abnormal psychological questionnaires and/or clinically indicated, psychology evaluation		X	X	X
If abnormally MOCA and/or clinically indicated, neurology visit		X	X	X
If abnormally WHODAS and/or clinically indicated, rehabilitation medicine visit		X	X	X

Legend: T1, T2 and T3: post COVID-19 visit at 2-3 months, 6-9 months and 12-15 months after clinical recovery.

Blood/serum samples collection: whole blood samples will be stored, centrifuged, and separated into plasma and peripheral blood mononuclear cells for immunological and viral analyses

WHO CRF, WHO’s Post COVID case report form; MRC dyspnoea scale, Modified Medical Research Council Dyspnoea Scale; QoL (EQ5D-5L), quality of life (5-level EQ-5D version); MOCA, Montreal Cognitive Assessment; HADS-A/D, Hospital Anxiety and Depression Scale; PCL-5, Post Traumatic Stress Disorders (PTSD) checklist for DSM-5; WHODAS, World Health Organization Disability Assessment schedule 2.0, 12-item version; DLCO, Diffusing capacity of the lung for carbon monoxide test

### Questionnaires administration

The fatigue numerical rating scale will be used to assess the severity of fatigue experienced by patients, while the MRC dyspnoea scale will be used to evaluate breathlessness. Quality of life will be assessed using the EQ5D-5L tool, which consists of the EQ-5D descriptive system and the EQ visual analogue scale (EQ-VAS). The HADS will be used to evaluate symptoms of anxiety and depression, while the PCL-5 will be used for first screening for PTSD and monitoring symptoms change during time. The MOCA will be used to assess cognitive function, and the WHODAS 2.0 will be used to measure health and disabilities in clinical practice. All questionnaires with the relative scores and the CRF will be listed in Table 2 and more detailed information attached as supplementary documents (Supplementary Appendix).

**Table 2:** Presenting the questionnaires administered to enrolled patients, along with information regarding their scoring

Administered questionnaires	
**Score**	**Levels/range**
Symptom assessment with COVID-19 WHO CRF	-Yes, but not present anymore
	-Yes, still present
	-Yes, intermittent
	-No
	-Unknown
Fatigue Numerical Rating Scale Fatigue Severity Scale	Likert Scale from 1 to 7
	Likert Scale from 0 to 10
Modified Medical Research Council (MRC) Dyspnoea Scale	Likert Scale from 0 to 4
Comorbidity relapse or new comorbidity	-Yes
	-No
	-If yes, type of comorbidity and date of diagnosis
Hospital re-admission	-Yes
	-No
	-If yes, reason and date of re-admission
QoL (EQ5D-5L)	Likert scale from 1 to 5
	Scale from 0 to 100
Hospital Anxiety and Depression Scale (HADS-A/D)	Score Depression from 0 to 21
	Score Anxiety from 0 to 21
	(0–7 normal, 8–10 borderline, 11–21 patological)
Post Traumatic Stress Disorders (PTSD) checklist for DSM-5 (PCL-5)	Score from 0 to 80
Montreal Cognitive Assessment (MOCA)	Score from 0 to 30 (adjusted for age and education) (≥ 26 no cognitive impairment; < 26 cognitive impairment
Functioning (WHODAS)	Score from 0 to 100

Legend: WHO CRF, WHO’s Post COVID case report form (constitutional, respiratory, cardiac, neurological, digestive, muscoloskeletal, ear/nose/throat, psychological, psychiatric symptoms; dermatologic, renal, endocrine, haematologic, autoimmune manifestations); MRC dyspnoea scale, Modified Medical Research Council Dyspnoea Scale; QoL (EQ5D-5L), quality of life (5-level EQ-5D version); MOCA, Montreal Cognitive Assessment; HADS-A/D, Hospital Anxiety and Depression Scale; PCL-5, Post Traumatic Stress Disorders (PTSD) checklist for DSM-5; WHODAS, World Health Organization Disability Assessment schedule 2.0, 12-item version

### Biological samples collection

The study protocol includes collection of biological samples for hospitalised COVID-19 patients during the acute phase. Blood samples and a nasopharyngeal swab will be collected at hospital admission for assessment of SARS-CoV-2 diagnostics, viral variant and viral sequencing, and cellular immunity. Blood samples will also be collected at different time points after enrolment in the post-COVID-19 cohort for the same analysis as in the acute phase. Whole blood samples will be stored, centrifuged, and separated into plasma, and peripheral blood mononuclear cells will be separated for immunological and viral analysis. Samples will be shipped to the University of Siena, Italy, for SARS-CoV-2 neutralising antibodies and for assessment of neutralisation assays, and to Karolinska Institutet, Sweden, for neutralisation assays and for assessment of SARS-CoV-2 diagnostics (WP2 of EuCare Project). The shipment of samples will follow specific regulations and procedures for packaging and international transport. All stored samples will be analysed and destroyed in accordance with current legal and ethical requirements.

### Data collection

During the acute phase of COVID-19 disease, data collection involves gathering demographic, clinical, and viro-immunological data. Subsequently, data collection is requested at each follow-up time point. To facilitate this process, a specially designed data management suite has been implemented. It incorporates a security-focused architecture that enables manual data entry into case report forms and secure file transfer for bulk data uploads. This flexible approach accommodates the various data infrastructures employed by the participating centres, whether they collect data from databases or manually. All data is meticulously curated and consolidated within a centralised relational database.

Data collection procedures strictly adhere to the established protocol, the most recent version of the Declaration of Helsinki, the International Conference on Harmonization Good Clinical Practice (ICH-GCP) or ISO EN 14155 guidelines, the regulation 2016/679 GDPR, as well as all relevant national legal and regulatory requirements.

### Data analysis

The study data will be presented as absolute numbers and percentages for categorical data, median and Interquartile Range or mean and standard deviation for quantitative variables. The incidence of post-COVID-19 condition will be calculated, and comparison between groups based on exposure factors of interest will be performed using appropriate statistical tests such as Student t-test or Mann Whitney and Chi-square test. Predictors of post COVID-19 condition will be investigated by fitting a univariable and multivariable logistic regression analysis which will be adjusted for possible confounders.

Due to potential losses to follow-up, not all hospitalised COVID-19 patients and patients diagnosed with SARS-CoV-2 infection will attend the post COVID-19 visit. This may result in a non-representative sample, potentially leading to collider bias and distorting the estimation of the effect of an exposure of interest. This bias can be addressed for hospitalised patients by using Inverse Probability Weighting through the creation of a pseudo-sample where individuals are weighted according to the inverse of the probability of being sampled for the post COVID-19 visit. A multivariable logistic regression model will be fitted to estimate the probability of COVID-19 hospitalised individuals to be included in the post COVID-19 cohort based on their characteristics (propensity score, PS), and weights inversely proportional to this probability will be generated (1/PS). Weighted logistic regression analyses will then be used to investigate predictors of post COVID-19 condition. Missing data will be handled using the missing “indicator method,” or alternatively using multiple imputations technique.

The sample size calculation will be determined by the number of expected events in the follow-up period. Based on the currently available data, we estimated an incidence of WHO post COVID-19 condition of 54% (95%CI 45–69%). We will use the comparison between the risk of developing post-COVID-19 condition in participants infected with the Delta versus Omicron viral variant of concern (VoC) for the sample size calculation. According to recent analysis, after controlling for vaccination and other confounders, the risk of hospitalisation comparing Omicron versus Delta variant ranged between 0.55 and 0.88 [21]. Our assumption is that if post COVID-19 condition occurs due to the direct effects of the virus (e.g., through disease severity, level of hyper-inflammation, or other), a similar difference could be seen when comparing the long-term risk of developing post COVID-19 condition by VoC. The cohort of 2,300 participants, accounting for 35% of follow-up, will still guarantee 90% power to detect a reduction in the risk of post-acute sequelae of at least 25% comparing participants infected with Omicron vs. Delta variants.

Data handling and statistical analyses will be performed by the team of statisticians and Artificial Intelligence experts in WP5. Python computing will be used for executing the statistical analysis.

## Study sponsor

The EuCARE-POSTCOVID study is an investigator-initiated study and has been initiated by the partners in EuCARE. The study is coordinated by ASST Santi Paolo and Carlo, Milan, Italy. The main coordinator is EuResist Network, a European Economic Interest Grouping based in Rome, Italy. The study is financed by EU Horizon Europe Research and Innovation Programme under Grant Agreement N° 101,046,016.

## Possible study strengths and limitations

The strengths of this medical study include its international cohort of patients who will be followed-up for one year after acute infection. The study also collects a comprehensive list of symptoms and objective measures of symptoms, such as dyspnoea, fatigue, and psychological issues, through standardised questionnaires and scales. Additionally, blood samples will be collected during the acute phase and follow-up for future studies on possible pathogenetic mechanisms of post COVID-19 condition.

However, there are also some potential limitations of the study to consider. One possible limitation is the risk of selection bias, as patients without ongoing symptoms may not keep follow-up appointments, leading to losses in follow-up, despite some statistical methods could be used to handle this bias. Another possible limitation is the potential overestimation of post COVID-19 condition due to the WHO definition, which includes any kind of symptom.

## Data dissemination

The results of this study, regardless of whether they are positive, negative, or inconclusive, will be published in peer-reviewed journals and/or presented at national and international conferences. Open access journals will be given preference for publication. All publications and presentations will be listed on the EuCARE webpage.

## Discussion

The EuCARE project comprises several patient cohorts and schools that provide an advanced response to COVID-19 epidemics. The EuCARE-POSTCOVID study is responsible for investigating the characteristics of the post COVID-19 condition, which seems to affect up to 10–15% of patients, according to a recent review [22]. Despite the current reduction in the incidence and severity of acute SARS-CoV-2 infections in Europe, the post-COVID-19 condition remains a medical challenge. The clinical course of the syndrome, its possible risk factors, and the best diagnostic-therapeutic approach are not yet entirely understood. First data shows that the symptoms could last for one year or more.

A large group of definitions has been proposed and adopted by different research groups to indicate the failure to return to a pre-COVID state of health. We will use the 2021 definition of post-COVID-19 condition identified by the WHO because it appears to be the most standardised and globally applicable definition so far. This definition will allow us to compare our data with those of other similar cohorts [13, 16, 23, 24]. Since the WHO definition includes any symptoms that don't have an alternative diagnosis, it is quite nonspecific and could cause an overestimation of COVID-19 sequelae. We will also focus on possible clusters of symptoms identifying phenotypes of post-COVID-19 condition. This study will focus on understanding the incidence and predictors of post-COVID-19 syndrome by comparing the different pandemic waves, thanks to the long duration of the study and the possibility of including retrospective data from the beginning of the epidemics.

Among predictors, the association between gender and post-COVID-19 syndrome will be explored. Our preliminary data and previous cohorts have underlined a possible higher prevalence of the condition in females, though the reason for this association is not known [13].

The comprehensive questionnaires for symptom collection and the scales for the objective and standardised measure of symptoms such as dyspnoea, fatigue, and psychological issues (anxiety, depression, PTSD, and health-related quality of life) will be repeated at different time points, allowing us to understand the duration and evolution of symptoms over time. Through questionnaires, data about vaccination against SARS CoV-2, reinfections, and new health problems requiring hospital admission or new diagnosis after COVID-19, such as the new onset of diabetes or other health conditions, will be collected.

The collection of biological samples will allow future studies on pathogenetic mechanisms of post-COVID-19 sequelae, such as persistent or excessive inflammation, immune dysregulation, or viral reservoirs.

The strengths of this study protocol are the inclusion of several cohorts outside Europe and the long study period that allows a clear and wide overview of the post COVID-19 condition. We also need to recognise that the study has some limitations, as already stated. Possible solutions to overcome these limitations could be the use of statistical methods such as propensity score matching to address biases due to losses of follow-up. The telehealth approach that is currently being implemented at the coordinating centre for performing post-COVID-19 evaluations without coming to the hospital could reduce losses to follow-up.

In conclusion, the EuCARE-POSTCOVID Study is an international multicentre cohort study that aims to better define and characterise the post COVID-19 condition over the different pandemic waves. The comprehension of the current epidemiology, predictors, clinical manifestations, and pathogenetic mechanisms of this syndrome is crucial in delineating the best management and therapeutic approaches that could improve patients' quality of life.

### Supplementary Information

**Additional file 1. **

## Data Availability

All data (dataset and/or analyses results during the current studies) will be available by request to corresponding author.

## Abbreviations

SARS-CoV-2: Severe acute respiratory syndrome coronavirus 2
COVID-19: Coronavirus disease 2019
PASC: Post-acute sequelae of COVID-19
WHO: World Health Organization
WP: Work package
VoC: Variant of concern
EuCARE: European cohorts of patients and schools to advance response to epidemics
CT: Computed tomography
MRC: Medical Research Council
EQ5D-5L: 5-Level EQ-5D version
EQ VAS: EQ visual analogue scale
HADS: Hospital anxiety and depression scale
PCL-5: Post-traumatic stress disorder checklist-5
MOCA: Montreal cognitive assessment;
WHODAS 2.0: WHO disability assessment schedule 2.0
CRF: Case report form
ICH-GCP: International conference on harmonization good clinical practice

## References

[1] WHO. WHO Coronavirus (COVID-19) Dashboard with Vaccination Data, updated March 2023. WHO. https://covid19.who.int/.

[2] Greenhalgh T, Knight M, A’Court C, Buxton M, Husain L. Management of post-acute covid-19 in primary care. BMJ. 2020;370. 10.1136/bmj.m3026. 10.1136/bmj.m3026 32784198

[3] Sivan M, Taylor S (2020). NICE guideline on long covid: Research must be done urgently to fill the many gaps in this new “living guideline”. BMJ.

[4] Haute Autorité De Santè, Réponses rapides dans le cadre de la COVID-19 Symptômes prolongés à la suite d’une Covid-19 de l’adulte - Diagnostic et prise en charge, méthode de réponse rapide, 19 janvier 2023; available at www.has-sante.fr.

[5] Cabrera Martimbianco AL, Pacheco RL, Bagattini ÂM, Riera R (2021). Frequency, signs and symptoms, and criteria adopted for long COVID-19: A systematic review. Int J Clin Pract.

[6] Huang C, Huang L, Wang Y (2021). 6-month consequences of COVID-19 in patients discharged from hospital: a cohort study. Lancet.

[7] Angelo Carfì, Roberto Bernabei FL. Persistent Symptoms in Patients After Acute COVID-19. JAMA Netw Open. Published online 2020. 10.1056/nejmp2014836. 10.1001/jama.2020.12603 PMC7349096 32644129

[8] Mayssam Nehme, Olivia Braillard, Delphine Courvoisier, Gabriel Alcoba, Sigiriya Aebischer Perone, François Chappuis IG. COVID-19 Symptoms: Longitudinal Evolution and Persistence in Outpatient Settings. Ann Intern Med. 2022;175(8):W81. 10.7326/L22-0211. 10.7326/M20-5926 PMC7741180 33284676

[9] Groff D, Sun A, Ssentongo AE (2021). Short-term and Long-term Rates of Postacute Sequelae of SARS-CoV-2 Infection: A Systematic Review. JAMA Netw Open.

[10] Claudia Carvalho-Schneider, Emeline Laurent, Adrien Lemaignen, Emilie Beaufils, Céline Bourbao-Tournois, Saïd Laribi, Thomas Flament, Nicole Ferreira-Maldent, Franck Bruyère, Karl Stefic, Catherine Gaudy-Graffin, Leslie Grammatico-Guillon LB. Follow-up of adults with noncritical COVID-19 two months after symptom onset. Clin Microbiol Infect. 2020;(January):1–7. 10.1016/j.cmi.2020.09.052 PMC7534895 33031948

[11] D’cruz RF, Waller MD, Perrin F (2021). Chest radiography is a poor predictor of respiratory symptoms and functional impairment in survivors of severe covid-19 pneumonia. ERJ Open Res.

[12] Goërtz YMJ, Van Herck M, Delbressine JM (2020). Persistent symptoms 3 months after a SARS-CoV-2 infection: The post-COVID-19 syndrome?. ERJ Open Res.

[13] Bai F, Tomasoni D, Falcinella C, et al, Female gender is associated with long COVID syndrome: a prospective cohort study. Clin Microbiol Infect. 2022;28(4):611.e9–611.e16. 10.1016/j.cmi.2021.11.002 PMC8575536 34763058

[14] Dubey A, Choudhary S, Kumar P, Tomar S. Emerging SARS-CoV-2 Variants: Genetic Variability and Clinical Implications. Curr Microbiol. 2022;79(1). 10.1007/s00284-021-02724-1. 10.1007/s00284-021-02724-1 PMC8669229 34905108

[15] Raimondi S, Gandini S, Rubio Quintanares GH (2023). European Cohorts of patients and schools to Advance Response to Epidemics (EuCARE): a cluster randomised interventional and observational study protocol to investigate the relationship between schools and SARS-CoV-2 infection. BMC Infect Dis.

[16] Sudre CH, Murray B, Varsavsky T (2021). Attributes and predictors of long COVID. Nat Med.

[17] Bergwerk M, Gonen T, Lustig Y (2021). Covid-19 Breakthrough Infections in Vaccinated Health Care Workers. N Engl J Med.

[18] Arévalos V, Ortega-Paz L, Rodríguez-Arias JJ, et al. Acute and chronic effects of COVID-19 on the cardiovascular system. J Cardiovasc Dev Dis. 2021;8(10). 10.3390/jcdd8100128. 10.3390/jcdd8100128 PMC8541609 34677197

[19] Seeble J, Waterboer T, Hippchen T (2022). Persistent Symptoms in Adult Patients 1 Year after Coronavirus Disease 2019 (COVID-19): A Prospective Cohort Study. Clin Infect Dis.

[20] Ferrucci R, Dini M, Groppo E (2021). Long-lasting cognitive abnormalities after COVID-19. Brain Sci.

[21] Ferguson N, Ghani A, Hinsley W, Volz E, College I. Report 50: Hospitalisation risk for Omicron cases in England. Imp Coll Rep. 2021;(December):1–12. 10.25561/93035%0A. https://www.imperial.ac.uk/media/imperial-college/medicine/mrc-gida/2021-12-22-COVID19-Report-50.pdf.

[22] Nalbandian A, Sehgal K, Gupta A (2021). Post-acute COVID-19 syndrome. Nat Med.

[23] Ghossein-Doha C, Wintjens MSJN, Janssen EBNJ (2022). Prevalence, pathophysiology, prediction and health-related quality of life of long COVID: Study protocol of the longitudinal multiple cohort CORona Follow Up (CORFU) study. BMJ Open.

[24] Xiong Q, Xu M, Li J (2021). Clinical sequelae of COVID-19 survivors in Wuhan, China: a single-centre longitudinal study. Clin Microbiol Infect.

