# Supplementary material for: EuCARE-POSTCOVID Study: a multicentre cohort study on long-term post-COVID-19 manifestations
Source: BMC Infect Dis. 2023 Oct 13;23:684. doi: 10.1186/s12879-023-08595-0 (PMC10576381; doi:10.1186/s12879-023-08595-0)
Supplement: Supplementary file 1 — Additional file 1. [file 12879_2023_8595_MOESM1_ESM.pdf]

SUPPLEMENTARY DOCUMENTS

THE POST-COVID-19 WORLD HEALTH ORGANIZATION (WHO) CASE REPORT FORM (CRF)

This is a short version of the post COVID-19 WHO CRF for collection of data on symptoms and newly emerged complaints in individuals recovering from COVID-19.

World Health Organization

2.6 Incidence of symptoms after acute illness of COVID-19

Did the participant experience any of the following symptoms after the acute illness of COVID-19/ since hospital discharge for COVID-19, that were **not experienced** before the acute episode of COVID-19? ☐Yes ☐No ☐Unknown;
If yes, please respond to questions below:
Anxiety: ☐Yes, but not present anymore ☐Yes, still present ☐Yes, intermittent ☐No ☐Unknown;
Behaviour change: ☐Yes, but not present anymore ☐Yes, still present ☐Yes, intermittent ☐No ☐Unknown;
Can't move and/or feel one side of body or face: ☐Yes, but not present anymore ☐Yes, still present ☐Yes, intermittent ☐No ☐Unknown;
Chest pain: ☐Yes, but not present anymore ☐Yes, still present ☐Yes, intermittent ☐No ☐Unknown;
Constipation: ☐Yes, but not present anymore ☐Yes, still present ☐Yes, intermittent ☐No ☐Unknown;
Depressed mood: ☐Yes, but not present anymore ☐Yes, still present ☐Yes, intermittent ☐No ☐Unknown;
Diarrhoea: ☐Yes, but not present anymore ☐Yes, still present ☐Yes, intermittent ☐No ☐Unknown;
Dysmenorrhea ☐Yes, but not present anymore ☐Yes, still present ☐Yes, intermittent ☐No ☐Unknown;
Dizziness/light headedness: ☐Yes, but not present anymore ☐Yes, still present ☐Yes, intermittent ☐No ☐Unknown;
Fainting/blackouts: ☐Yes, but not present anymore ☐Yes, still present ☐Yes, intermittent ☐No ☐Unknown;
Fever: ☐Yes, but not present anymore ☐Yes, still present ☐Yes, intermittent ☐No ☐Unknown;
Forgetfulness: ☐Yes, but not present anymore ☐Yes, still present ☐Yes, intermittent ☐No ☐Unknown;
Jerking of limbs: ☐Yes, but not present anymore ☐Yes, still present ☐Yes, intermittent ☐No ☐Unknown;
Joint pain/swelling: ☐Yes, but not present anymore ☐Yes, still present ☐Yes, intermittent ☐No ☐Unknown;
Loss of appetite: ☐Yes, but not present anymore ☐Yes, still present ☐Yes, intermittent ☐No ☐Unknown;
Loss of interest/pleasure: ☐Yes, but not present anymore ☐Yes, still present ☐Yes, intermittent ☐No ☐Unknown;
Lumpy lesions: (purple/pink/bluish) **on toes/COVID toes:** ☐Yes, but not present anymore ☐Yes, still present ☐Yes, intermittent ☐No ☐Unknown;
Nausea/vomiting: ☐Yes, but not present anymore ☐Yes, still present ☐Yes, intermittent ☐No ☐Unknown;
Numbness or tingling: ☐Yes, but not present anymore ☐Yes, still present ☐Yes, intermittent ☐No ☐Unknown;
Pain on breathing: ☐Yes, but not present anymore ☐Yes, still present ☐Yes, intermittent ☐No ☐Unknown;
Palpitations: ☐Yes, but not present anymore ☐Yes, still present ☐Yes, intermittent ☐No ☐Unknown;
Persistent dry cough: ☐Yes, but not present anymore ☐Yes, still present ☐Yes, intermittent ☐No ☐Unknown;
Persistent fatigue: ☐Yes, but not present anymore ☐Yes, still present ☐Yes, intermittent ☐No ☐Unknown;
Problems hearing: ☐Yes, but not present anymore ☐Yes, still present ☐Yes, intermittent ☐No ☐Unknown;
Persistent headache: ☐Yes, but not present anymore ☐Yes, still present ☐Yes, intermittent ☐No ☐Unknown;
Persistent muscle pain: ☐Yes, but not present anymore ☐Yes, still present ☐Yes, intermittent ☐No ☐Unknown;
Post-exertional malaise: ☐Yes, but not present anymore ☐Yes, still present ☐Yes, intermittent ☐No ☐Unknown;
Problems passing urine: ☐Yes, but not present anymore ☐Yes, still present ☐Yes, intermittent ☐No ☐Unknown;
Problems seeing: ☐Yes, but not present anymore ☐Yes, still present ☐Yes, intermittent ☐No ☐Unknown;
Problem swallowing: ☐Yes, but not present anymore ☐Yes, still present ☐Yes, intermittent ☐No ☐Unknown;
Problems with balance: ☐Yes, but not present anymore ☐Yes, still present ☐Yes, intermittent ☐No ☐Unknown;
Problems with gait/falls: ☐Yes, but not present anymore ☐Yes, still present ☐Yes, intermittent ☐No ☐Unknown;
Reduced smell: ☐Yes, but not present anymore ☐Yes, still present ☐Yes, intermittent ☐No ☐Unknown;
Reduced taste: ☐Yes, but not present anymore ☐Yes, still present ☐Yes, intermittent ☐No ☐Unknown;
Ringing in ears: ☐Yes, but not present anymore ☐Yes, still present ☐Yes, intermittent ☐No ☐Unknown;
Seizures: ☐Yes, but not present anymore ☐Yes, still present ☐Yes, intermittent ☐No ☐Unknown;
Shortness of breath: ☐Yes, but not present anymore ☐Yes, still present;
If yes: ☐Present ☐ At rest ☐With activity; ☐Yes, intermittent ☐No ☐Unknown;
Skin rash: ☐Yes, but not present anymore ☐Yes, still present ☐Yes, intermittent ☐No ☐Unknown;
If yes, please tick all areas of the body that apply: ☐Face ☐Trunk (stomach or back) ☐Arms ☐Legs ☐Buttocks ☐Toes ☐Fingers;
Slowness of movement: ☐Yes, but not present anymore ☐Yes, still present ☐Yes, intermittent ☐No ☐Unknown;
Sleeping less: ☐Yes, but not present anymore ☐Yes, still present ☐Yes, intermittent ☐No ☐Unknown;
Sleeping more: ☐Yes, but not present anymore ☐Yes, still present ☐Yes, intermittent ☐No ☐Unknown;
Stiffness of muscles: ☐Yes, but not present anymore ☐Yes, still present ☐Yes, intermittent ☐No ☐Unknown;
Stomach pain: ☐Yes, but not present anymore ☐Yes still present ☐Yes, intermittent ☐No ☐Unknown;
Swollen ankles: ☐Yes, but not present anymore ☐Yes, still present ☐Yes, intermittent ☐No ☐Unknown;
Tremors: ☐Yes, but not present anymore ☐Yes, still present ☐Yes, intermittent ☐No ☐Unknown;
Trouble in concentrating: ☐Yes, but not present anymore ☐Yes, still present ☐Yes, intermittent ☐No ☐Unknown;
Weakness in limbs: ☐Yes, but not present anymore ☐Yes, still present ☐Yes, intermittent ☐No ☐Unknown;
Weight loss: ☐Yes, but not present anymore ☐Yes, still present ☐Yes, intermittent ☐No ☐Unknown;
The following questions should not be completed for children <15yrs:
Erectile dysfunction: ☐Yes, but not present anymore ☐Yes, still present ☐Yes, intermittent ☐No ☐Unknown;
Hallucinations (seeing or hearing things others don't see or hear): ☐Yes, but not present anymore ☐Yes, still present ☐Yes, intermittent ☐No ☐Unknown

WORLD HEALTH ORGANIZATION DISABILITY ASSESSMENT SCHEDULE 2.0 (WHODAS 2.0)

The WHODAS 2.0 is a practical and comprehensive assessment tool designed to measure health and disabilities in clinical settings. It assesses the level of functioning across six domains, including Cognitive Activities, Mobility, Self-Care, Interpersonal Relationships, Activities of Daily Living, and Participation. These domains capture a wide range of functional abilities and limitations.

WHODAS 2.0

WORLD HEALTH ORGANIZATION
DISABILITY ASSESSMENT SCHEDULE 2.0

12-item version, self-administered

This questionnaire asks about difficulties due to health conditions. Health conditions include diseases or illnesses, other health problems that may be short or long lasting, injuries, mental or emotional problems, and problems with alcohol or drugs.

Think back over the past 30 days and answer these questions, thinking about how much difficulty you had doing the following activities. For each question, please circle only one response.

In the past 30 days, how much difficulty did you have in:						
S1	Standing for long periods such as 30 minutes?	None	Mild	Moderate	Severe	Extreme or cannot do
S2	Taking care of your household responsibilities?	None	Mild	Moderate	Severe	Extreme or cannot do
S3	Learning a new task, for example, learning how to get to a new place?	None	Mild	Moderate	Severe	Extreme or cannot do
S4	How much of a problem did you have joining in community activities (for example, festivities, religious or other activities) in the same way as anyone else can?	None	Mild	Moderate	Severe	Extreme or cannot do
S5	How much have you been emotionally affected by your health problems?	None	Mild	Moderate	Severe	Extreme or cannot do

Please continue to next page...

.

In the past 30 days, how much difficulty did you have in:						
S6	Concentrating on doing something for ten minutes?	None	Mild	Moderate	Severe	Extreme or cannot do
S7	Walking a long distance such as a kilometre [or equivalent]?	None	Mild	Moderate	Severe	Extreme or cannot do
S8	Washing your whole body?	None	Mild	Moderate	Severe	Extreme or cannot do
S9	Getting dressed?	None	Mild	Moderate	Severe	Extreme or cannot do
S10	Dealing with people you do not know?	None	Mild	Moderate	Severe	Extreme or cannot do
S11	Maintaining a friendship?	None	Mild	Moderate	Severe	Extreme or cannot do
S12	Your day-to-day work?	None	Mild	Moderate	Severe	Extreme or cannot do

H1	Overall, in the past 30 days, how many days were these difficulties present?	***Record number of days*** ____
H2	In the past 30 days, for how many days were you totally unable to carry out your usual activities or work because of any health condition?	***Record number of days*** ____
H3	In the past 30 days, not counting the days that you were totally unable, for how many days did you cut back or reduce your usual activities or work because of any health condition?	***Record number of days*** ____

This completes the questionnaire. Thank you.

FATIGUE SEVERITY SCALE (FSS)

The fatigue numerical rating scale is a single-item Patient Reported Outcome (PRO) measure assessing severity of fatigue. It is a 11-point horizontal scale with 0 representing “no fatigue” and 10 representing “as bad as you can imagine”. Patients are asked to rate their fatigue by selecting the number that describes the worst level of fatigue during the past 24 hours.

FATIGUE SEVERITY SCALE (FSS)

Date ______________________________ Name __________________________________

Please circle the number between 1 and 7 which you feel best fits the following statements. This refers to your usual way of life within the last week. 1 indicates "strongly disagree" and 7 indicates "strongly agree."

Read and circle a number.	Strongly Disagree → Strongly Agree						
1. My motivation is lower when I am fatigued.	1	2	3	4	5	6	7
2. Exercise brings on my fatigue.	1	2	3	4	5	6	7
3. I am easily fatigued.	1	2	3	4	5	6	7
4. Fatigue interferes with my physical functioning.	1	2	3	4	5	6	7
5. Fatigue causes frequent problems for me.	1	2	3	4	5	6	7
6. My fatigue prevents sustained physical functioning.	1	2	3	4	5	6	7
7. Fatigue interferes with carrying out certain duties and responsibilities.	1	2	3	4	5	6	7
8. Fatigue is among my most disabling symptoms.	1	2	3	4	5	6	7
9. Fatigue interferes with my work, family, or social life.	1	2	3	4	5	6	7

VISUAL ANALOGUE FATIGUE SCALE (VAFS)

Please mark an "X" on the number line which describes your global fatigue with 0 being worst and 10 being normal.

0	1	2	3	4	5	6	7	8	9	10

POST-TRAUMATIC STRESS DISORDER (PTSD) CHECKLIST FOR DSM-5 (PCL-5)

The PCL-5 is a 20-item self-report measure that assesses the 20 DSM-5 symptoms of PTSD with a 5-point scale for each symptom. The PCL-5 is used for first screening for PTSD and monitoring symptoms change during time. A total symptom severity score (ranging from 0 to 80) can be obtained and a cut-off of 31-33 could be used as indicative of PTSD. Items can be also summarized in 4 different clusters, and a provisional PTSD diagnosis can be made analysing results of single clusters according to DSM-5 diagnostic rules.

National Center for PTSD
POSTTRAUMATIC STRESS DISORDER

PTSD Checklist for *DSM-5* (PCL-5)

Version date: 11 April 2018

Reference: Weathers, F. W., Litz, B. T., Keane, T. M., Palmieri, P. A., Marx, B. P., & Schnurr, P. P. (2013). *The PTSD Checklist for DSM-5 (PCL-5) – Standard* [Measurement instrument]. Available from https://www.ptsd.va.gov/

URL: https://www.ptsd.va.gov/professional/assessment/adult-sr/ptsd-checklist.asp

Note: This is a fillable form. You may complete it electronically.

PCL-5

Instructions: Below is a list of problems that people sometimes have in response to a very stressful experience. Please read each problem carefully and then circle one of the numbers to the right to indicate how much you have been bothered by that problem in the past month.

In the past month, how much were you bothered by:	Not at all	A little bit	Moderately	Quite a bit	Extremely
1. Repeated, disturbing, and unwanted memories of the stressful experience?	0	1	2	3	4
2. Repeated, disturbing dreams of the stressful experience?	0	1	2	3	4
3. Suddenly feeling or acting as if the stressful experience were actually happening again (as if you were actually back there reliving it)?	0	1	2	3	4
4. Feeling very upset when something reminded you of the stressful experience?	0	1	2	3	4
5. Having strong physical reactions when something reminded you of the stressful experience (for example, heart pounding, trouble breathing, sweating)?	0	1	2	3	4
6. Avoiding memories, thoughts, or feelings related to the stressful experience?	0	1	2	3	4
7. Avoiding external reminders of the stressful experience (for example, people, places, conversations, activities, objects, or situations)?	0	1	2	3	4
8. Trouble remembering important parts of the stressful experience?	0	1	2	3	4
9. Having strong negative beliefs about yourself, other people, or the world (for example, having thoughts such as: I am bad, there is something seriously wrong with me, no one can be trusted, the world is completely dangerous)?	0	1	2	3	4
10. Blaming yourself or someone else for the stressful experience or what happened after it?	0	1	2	3	4
11. Having strong negative feelings such as fear, horror, anger, guilt, or shame?	0	1	2	3	4
12. Loss of interest in activities that you used to enjoy?	0	1	2	3	4
13. Feeling distant or cut off from other people?	0	1	2	3	4
14. Trouble experiencing positive feelings (for example, being unable to feel happiness or have loving feelings for people close to you)?	0	1	2	3	4
15. Irritable behavior, angry outbursts, or acting aggressively?	0	1	2	3	4
16. Taking too many risks or doing things that could cause you harm?	0	1	2	3	4
17. Being "superalert" or watchful or on guard?	0	1	2	3	4
18. Feeling jumpy or easily startled?	0	1	2	3	4
19. Having difficulty concentrating?	0	1	2	3	4
20. Trouble falling or staying asleep?	0	1	2	3	4

MONTREAL COGNITIVE ASSESSMENT (MOCA)

The MOCA is a cognitive screening test consisting of 11 individual tests. The final score is obtained by summing the scores from each test. If the patient has less than 12 years of education, an additional point is added to the final score. A score below 26 is considered pathological, indicating potential cognitive impairment. The MOCA is designed to assess various cognitive domains and provide a measure of cognitive function, with lower scores indicating a greater likelihood of cognitive impairment.

MONTREAL COGNITIVE ASSESSMENT (MOCA)

NAME :
Education :
Sex :
Date of birth :
DATE :

VISUOSPATIAL / EXECUTIVE			POINTS
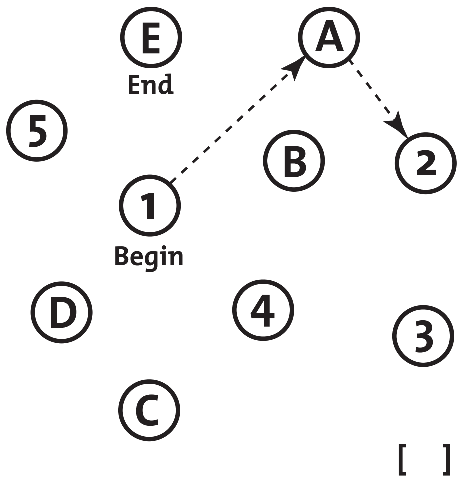 [ ]	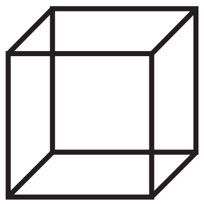 Copy cube [ ]	Draw CLOCK (Ten past eleven) (3 points) [ ] Contour [ ] Numbers [ ] Hands	__/5

NAMING			
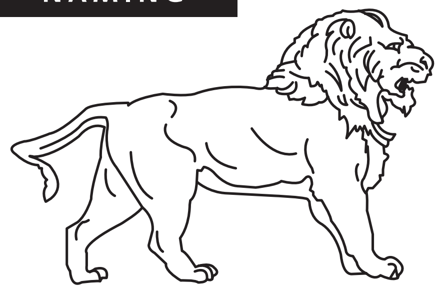 [ ]	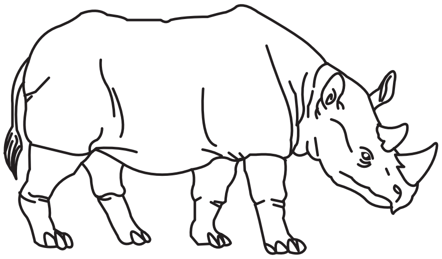 [ ]	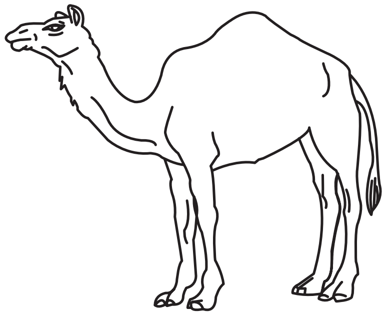 [ ]	__/3

MEMORY

Read list of words, subject must repeat them. Do 2 trials. Do a recall after 5 minutes.

	FACE	VELVET	CHURCH	DAISY	RED	No points
1st trial						
2nd trial						

ATTENTION

Read list of digits (1 digit/ sec.).	Subject has to repeat them in the forward order [ ] 2 1 8 5 4<br>Subject has to repeat them in the backward order [ ] 7 4 2	__/2
Read list of letters. The subject must tap with his hand at each letter A. No points if ≥ 2 errors	[ ] F B A C M N A A J K L B A F A K D E A A A J A M O F A A B	__/1
Serial 7 subtraction starting at 100	[ ] 93 [ ] 86 [ ] 79 [ ] 72 [ ] 65<br>4 or 5 correct subtractions: **3 pts**, 2 or 3 correct: **2 pts**, 1 correct: **1 pt**, 0 correct: **0 pt**	__/3

LANGUAGE

Repeat : I only know that John is the one to help today. [ ]<br>The cat always hid under the couch when dogs were in the room. [ ]	__/2
Fluency / Name maximum number of words in one minute that begin with the letter F [ ] ____ (N ≥ 11 words)	__/1

ABSTRACTION

Similarity between e.g. banana - orange = fruit [ ] train – bicycle [ ] watch - ruler	__/2

DELAYED RECALL

	Has to recall words WITH NO CUE	FACE [ ]	VELVET [ ]	CHURCH [ ]	DAISY [ ]	RED [ ]	Points for UNCUED recall only	__/5
Optional	Category cue							
	Multiple choice cue							

ORIENTATION

[ ] Date [ ] Month [ ] Year [ ] Day [ ] Place [ ] City	__/6

© Z.Nasreddine MD Version November 7, 2004

www.mocatest.org

Normal ≥ 26 / 30

TOTAL __/30

Add 1 point if ≤ 12 yr edu

HOSPITAL ANXIETY AND DEPRESSION SCALE (HADS)

HADS includes 7 questions for anxiety and 7 questions for depression. A total HADS score between 8 and 10 denote “possible” cases and scores of 11 or more denoted “probable” cases in both the anxiety and depression scales. Thus, a score higher than 10 was used to identify symptoms of anxiety and depression.

Hospital Anxiety and Depression Scale (HADS)

Tick the box beside the reply that is closest to how you have been feeling in the past week.
Don’t take too long over you replies: your immediate is best.

D	A		D	A	
		**I feel tense or 'wound up':**			**I feel as if I am slowed down:**
	3	Most of the time	3		Nearly all the time
	2	A lot of the time	2		Very often
	1	From time to time, occasionally	1		Sometimes
	0	Not at all	0		Not at all
		**I still enjoy the things I used to enjoy:**			**I get a sort of frightened feeling like 'butterflies' in the stomach:**
0		Definitely as much		0	Not at all
1		Not quite so much		1	Occasionally
2		Only a little		2	Quite Often
3		Hardly at all		3	Very Often
		**I get a sort of frightened feeling as if something awful is about to happen:**			**I have lost interest in my appearance:**
	3	Very definitely and quite badly	3		Definitely
	2	Yes, but not too badly	2		I don't take as much care as I should
	1	A little, but it doesn't worry me	1		I may not take quite as much care
	0	Not at all	0		I take just as much care as ever
		**I can laugh and see the funny side of things:**			**I feel restless as I have to be on the move:**
0		As much as I always could		3	Very much indeed
1		Not quite so much now		2	Quite a lot
2		Definitely not so much now		1	Not very much
3		Not at all		0	Not at all
		**Worrying thoughts go through my mind:**			**I look forward with enjoyment to things:**
	3	A great deal of the time	0		As much as I ever did
	2	A lot of the time	1		Rather less than I used to
	1	From time to time, but not too often	2		Definitely less than I used to
	0	Only occasionally	3		Hardly at all
		**I feel cheerful:**			**I get sudden feelings of panic:**
3		Not at all		3	Very often indeed
2		Not often		2	Quite often
1		Sometimes		1	Not very often
0		Most of the time		0	Not at all
		**I can sit at ease and feel relaxed:**			**I can enjoy a good book or radio or TV program:**
	0	Definitely	0		Often
	1	Usually	1		Sometimes
	2	Not Often	2		Not often
	3	Not at all	3		Very seldom

Please check you have answered all the questions

Scoring:
Total score: Depression (D) ___________ Anxiety (A) _____________
0-7 = Normal
8-10 = Borderline abnormal (borderline case)
11-21 = Abnormal (case)

HEALTH QUESTIONNAIRE (EQ-5D-5L)

The 5-level EQ-5D version (EQ5D-5L) is a standardised tool to assess quality of life. It consists of the EQ-5D descriptive system and the EQ visual analogue scale (EQ VAS). The descriptive system includes five dimensions: mobility, self-care, usual activities, pain/discomfort and anxiety/depression. Each dimension has 5 levels: no problems, slight, moderate, severe and extreme problems. The patient is asked to indicate his/her health state by ticking the box next to the most appropriate statement in each of the five dimensions. This decision results in a 1-digit number and the digits for the five dimensions can be combined into a 5-digit number that describes the patient's health state. The EQ VAS consists of a vertical visual analogue scale, where the endpoints are "The best health you can imagine" and "The worst health you can imagine".

Health Questionnaire (EQ-5D-5L)

Under each heading, please tick the ONE box that best describes your health TODAY.

MOBILITY

☐1 I have no problems in walking about
☐2 I have slight problems in walking about
☐3 I have moderate problems in walking about
☐4 I have severe problems in walking about
☐5 I am unable to walk about

SELF-CARE

☐1 I have no problems washing or dressing myself
☐2 I have slight problems washing or dressing myself
☐3 I have moderate problems washing or dressing myself
☐4 I have severe problems washing or dressing myself
☐5 I am unable to wash or dress myself

USUAL ACTIVITIES *(e.g. work, study, housework, family or leisure activities)*

☐1 I have no problems doing my usual activities
☐2 I have slight problems doing my usual activities
☐3 I have moderate problems doing my usual activities
☐4 I have severe problems doing my usual activities
☐5 I am unable to do my usual activities

PAIN / DISCOMFORT

☐1 I have no pain or discomfort
☐2 I have slight pain or discomfort
☐3 I have moderate pain or discomfort
☐4 I have severe pain or discomfort
☐5 I have extreme pain or discomfort

ANXIETY / DEPRESSION

☐1 I am not anxious or depressed
☐2 I am slightly anxious or depressed
☐3 I am moderately anxious or depressed
☐4 I am severely anxious or depressed
☐5 I am extremely anxious or depressed

UK (English) © 2009 EuroQol Group EQ-5D™ is a trade mark of the EuroQol Group

Health Questionnaire (EQ-5D-5L)

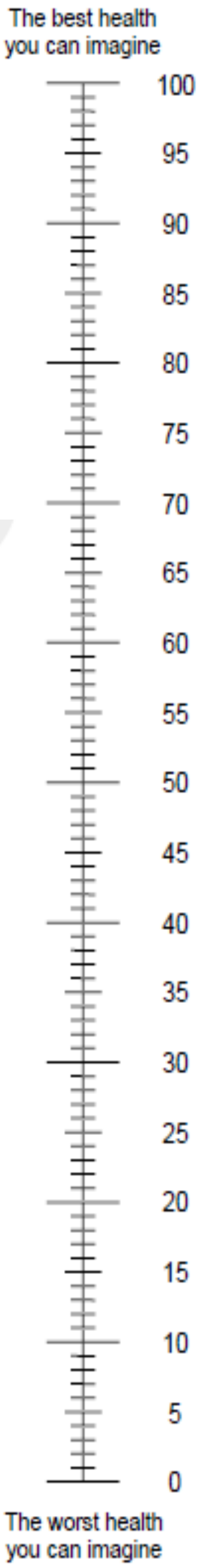

- We would like to know how good or bad your health is **TODAY**.
- This scale is numbered from 0 to 100.
- 100 means the best health you can imagine.
  0 means the worst health you can imagine.
- Mark an X on the scale to indicate how your health is **TODAY**
- Now, please write the number you marked on the scale in the

  below.

YOUR HEALTH TODAY =

UK (English) © 2009 EuroQol Group EQ-5D™ is a trade mark of the EuroQol Group

MODIFIED MEDICAL RESEARCH COUNCIL DYSPNOEA SCALE

The (MRC) dyspnoea scale is a questionnaire that consists of five statements about perceived breathlessness: grade 1, “I only get breathless with strenuous exercise”; grade 2, “I get short of breath when hurrying on the level or up a slight hill”; grade 3, “I walk slower than people of the same age on the level because of breathlessness or have to stop for breath when walking at my own pace on the level”; grade 4, “I stop for breath after walking 100 yards or after a few minutes on the level”; grade 5, “I am too breathless to leave the house”. Patients selected the grade that applied to them.

Bronchiectasis Toolbox

Modified Medical Research Council Dyspnoea Scale

0	"I only get breathless with strenuous exercise"
1	"I get short of breath when hurrying on the level or walking up a slight hill"
2	"I walk slower than people of the same age on the level because of breathlessness or have to stop for breath when walking at my own pace on the level"
3	"I stop for breath after walking about 100 yards or after a few minutes on the level"
4	"I am too breathless to leave the house" or "I am breathless when dressing"

Doherty DE et al. COPD: Consensus Recommendations for early diagnosis and treatment. Journal of Family Practice, Nov 2006
